# Caecal Volvulus Presenting as the Obstructed Right Inguinal Hernia

**DOI:** 10.7759/cureus.16264

**Published:** 2021-07-08

**Authors:** Mahalingam Sudharshan, Oseen Shaikh, Rajapriyan Panneerselvam, Uday Kumbhar, Naveen Kumar Gaur

**Affiliations:** 1 Surgery, Jawaharlal Institute of Postgraduate Medical Education and Research, Puducherry, IND

**Keywords:** caecal volvulus, strangulated hernia, herniorrhaphy, inguinal hernia, intestinal obstruction

## Abstract

Caecal volvulus is an uncommon cause of intestinal obstruction with varied clinical presentation. Surgical intervention without delay is considered the gold standard in its management. Strangulated inguinal hernia with caecal volvulus is a rarity. We report a case of a 55-year-old male with a history of long-standing right inguinal hernia, presented with the irreducibility of the hernia along with pain for one day. Clinically patient was diagnosed to have an obstructed inguinal hernia. On exploration, we found that there was a caecal volvulus in the hernia sac along with gangrene of the distal ileum, redundant sigmoid, and ascending colon. Resection and anastomosis of the gangrenous segment of the bowel were done along with herniorrhaphy and sigmoidopexy. Postoperatively patient improved without any complication.

## Introduction

Colonic volvulus is an axial twisting of a segment of the colon around its mesentery. Classical caecal volvulus is the clockwise, axial twisting of the terminal ileum, caecum, and ascending colon around its mesentery. It is an uncommon cause of intestinal obstruction [1]. In general, caecal volvulus has a varied clinical spectrum ranging from recurrent intermittent pain to acute fulminant intestinal obstruction. Caecal volvulus in a strangulated inguinal hernia is very rare. Surgical intervention without delay is considered the gold standard in its management [2].

## Case presentation

A 55-year-old male presented with a history of right-sided uncomplicated inguinoscrotal hernia for 15 years, which became irreducible for the last 6 h before presentation. The patient had mild pain over the swelling since it became irreducible. He also had two episodes of non-bilious, non-projectile vomiting. Examination of the right inguinoscrotal region revealed an irreducible swelling of size 15 cm x 12 cm reaching up to the base of the scrotum, without cough impulse and a buried penis. There was tenderness over the inguinal region. His lower abdomen was distended without any abdominal signs.

**Figure 1:**
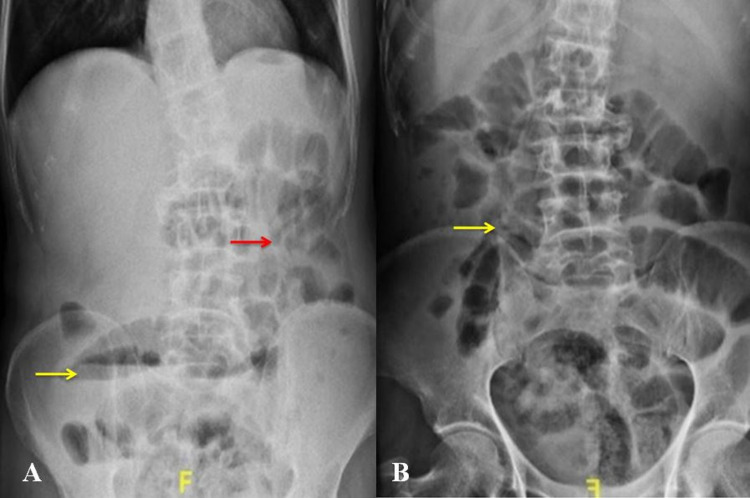
Plain radiograph of the abdomen showing (A) single air-fluid level (yellow arrow) with dilated small bowel loops (red arrow) and (B) few dilated bowel loops (arrow).

Haematological, biochemical, and arterial blood gas analyses were within normal limits. Plain radiographs of the abdomen showed multiple air-fluid levels with dilated small bowel loops confined to the left side of the abdomen (Figure 1).

**Figure 2:**
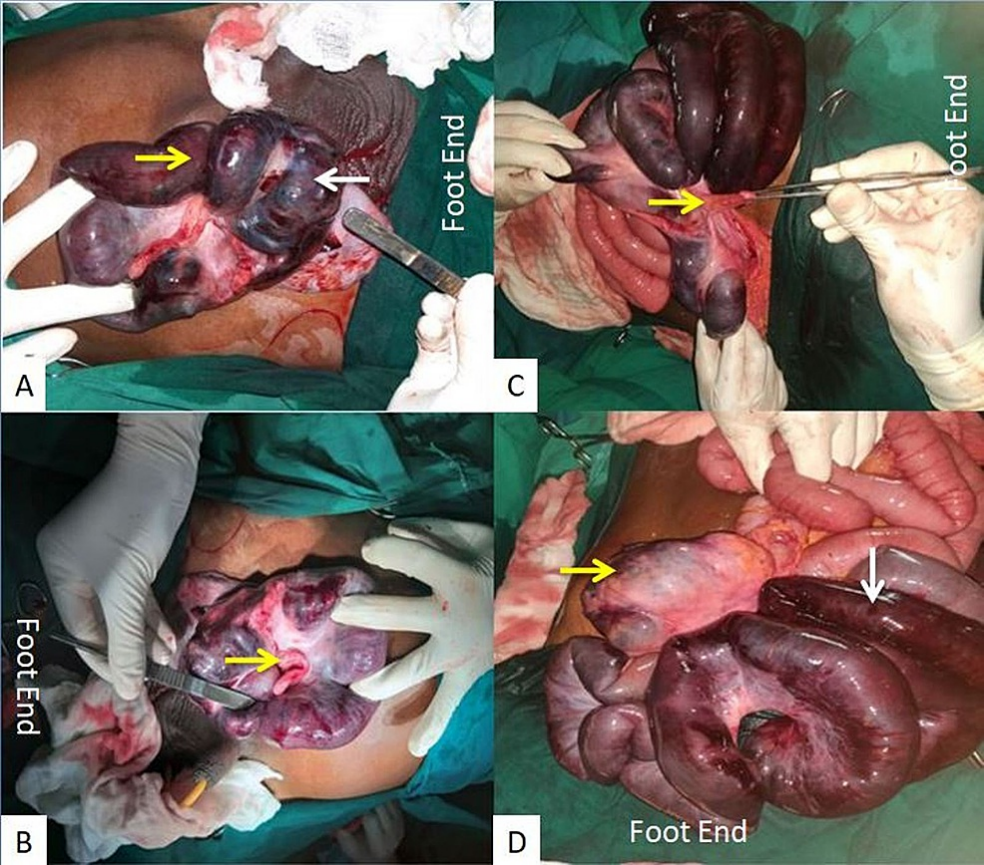
Intraoperative image showing (A) opened scrotal sac containing gangrenous caecum (white arrow) and ileum (yellow arrow) in the indirect inguinal hernia, (B) opened sac with caecum, appendix (arrow), and ileum, (C) derotated bowel loops with appendix (yellow arrow), and (D) derotated gangrenous ileum (white arrow) and ascending colon (yellow arrow).

The patient was diagnosed with an obstructed right inguinal hernia. Hence, emergency inguinoscrotal exploration was done. Once the hernial sac was opened, it was surprising that the sac contained caecum, appendix, and ileum, which were gangrenous. Hence, we proceeded with a lower midline laparotomy. We found a volvulus of 100 cm of the terminal ileum, caecum, and part of ascending colon. The whole of the volvular component was herniating through the deep ring into the inguinal canal (Figure 2).

**Figure 3:**
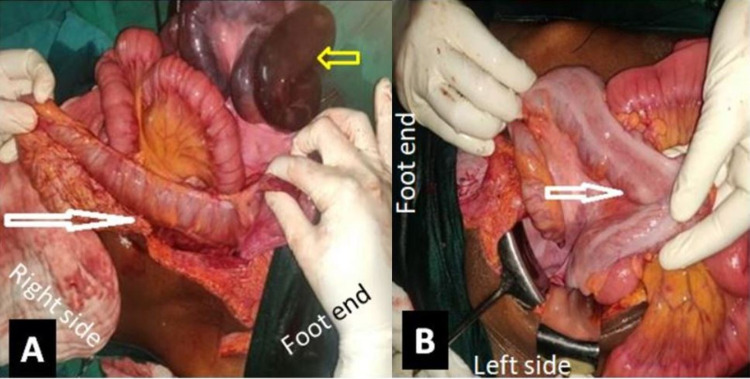
Intraoperative image showing (A) hypermobile and redundant ascending colon (white arrow) with gangrenous bowel loops (yellow arrow) and (B) redundant sigmoid colon (arrow).

Ascending colon, hepatic flexure, and sigmoid colon were hypermobile and redundant with long mesentery (Figure 3).

Right hemicolectomy with resection of the gangrenous bowel loops with covering loop ileostomy was done. The redundant sigmoid colon was fixed by sigmoidopexy. High ligation of the hernial sac along with Lytle’s and modified Basini’s repair was done. The postoperative period was uneventful without any complications.

## Discussion

The caecal volvulus is an axial twisting of the terminal ileum, caecum, and ascending colon around its mesentery. It is a rare cause of intestinal obstruction. However, it is the second most common colonic volvulus [2]. There are various predisposing factors, such as previous abdominal surgery, late-term pregnancy, adynamic ileus, chronic constipation, distal colonic obstruction, and high fiber intake, contributing to caecal volvulus. The most widely accepted and frequent factor is the non-peritonealization of the right colon (mobile caecum syndrome). 

Obstructed indirect inguinal hernias are commonly encountered in clinical practice, but a caecal volvulus presenting as an obstructed hernia is rare. Reznichenko et al. reported a case of caecal volvulus in a giant ventral hernia where repeated abdominal surgeries were a predisposing factor for the herniating caecum to undergo torsion due to progressive loss of its fixation [3]. Although most of the caecal volvulus is due to previous abdominal surgery, our patient did not have any history of abdominal surgery [4]. NeMoyer et al. reported a case of caecal volvulus in left-sided inguinal hernia with loss of domain causing closed-loop obstruction and perforation, where the abnormal axial rotation of the bowel could be attributed to loss of domain [4]. However, on the other hand, in our patient, the caecum and ascending colon were hypermobile, wherein the elongated mesentery must have facilitated the herniation of the twisting component.

A wide spectrum of presentation of caecal volvulus ranging from recurrent intermittent patterns to acute fulminant intestinal obstruction has been described. The clinical features may include recurrent abdominal pain and abdominal distension with the resolution of pain on passing flatus as in mobile caecal syndrome. Some patients present with acute fulminant intestinal obstruction. Surprisingly, in our case, the patient was haemodynamically stable with pain and tenderness limited to the inguinal swelling without any features of generalized peritonitis.

Although laboratory investigations are seldom diagnostic in a case of caecal volvulus, an elevated leukocyte count, lactate levels, and acidosis can differentiate it from strangulation. As all these parameters were within normal limits in our patient, a differential of strangulation was not considered preoperatively.

Radiological investigations are crucial for the diagnosis of caecal volvulus. A plain radiograph of the abdomen may reveal air-fluid levels, small bowel dilatation, and absence of gas in the distal colon in cases of caecal volvulus [2]. Contrast-enhanced computed tomography (CT) of the abdomen is superior [5]. It may reveal various signs, such as whirl sign, coffee bean sign, and split wall sign, specific for caecal volvulus [6]. We did not consider caecal volvulus as a differential diagnosis in our scenario with non-specific radiological findings in the background of obstructed inguinal hernia. Hence, CT was not performed, and the patient was taken immediately for emergency surgery. 

Although various treatment modalities are described in the literature, surgical intervention is superior and most effective. Manual detorsion with caecopexy, caecostomy, and right hemicolectomy is the surgical intervention available. In a viable bowel, a relatively safe procedure would be manual detorsion and caecopexy, but it has a higher recurrence rate, local complications, and poor long-term outcomes. On the contrary, when a non-viable or perforated bowel is encountered, resection and anastomosis of the bowel should be considered. The extent of resection depends upon the viability of the bowel loop, and hence surgical procedure should be individualized according to the patient. We performed resection of gangrenous ileal loops along with right hemicolectomy. Restoration of bowel continuity was achieved with ileotransverse anastomosis. Also, the patient had a redundant sigmoid colon where sigmoidopexy was done to prevent future volvulus.

## Conclusions

Patients with right inguinal hernias rarely contain caecum, ileum, and ascending colon. Volvulus of the caecum and terminal ileum presenting as the right inguinal hernia is an infrequent presentation. Clinicians must be aware of such a rare possibility. Patients with an obstructed inguinal hernia should be immediately taken to emergency surgery, considering the possibility of strangulation within. Patients with caecal volvulus should undergo a right hemicolectomy along with resection of the gangrenous bowel. To the best of our knowledge, this is the first case of caecal volvulus reported in the literature, presenting in the right inguinal hernia, occurring due to a redundant colon.
